# Social dominance influences individual susceptibility to an evolutionary trap in mosquitofish

**DOI:** 10.1002/eap.3081

**Published:** 2025-01-20

**Authors:** Lea Pollack, Michael Culshaw‐Maurer, Andrew Sih

**Affiliations:** ^1^ Department of Environmental Science and Policy University of California, Davis Davis California USA; ^2^ Department of Ecology, Evolution and Behavior University of Minnesota Saint Paul Minnesota USA; ^3^ Department of Ecology and Evolution University of California, Davis Davis California USA; ^4^ Metro Transit Minneapolis Minnesota USA

**Keywords:** dominance hierarchy, evolutionary trap, group size, microplastics, mosquitofish

## Abstract

Plastic pollution threatens almost every ecosystem in the world. Critically, many animals consume plastic, in part because plastic particles often look or smell like food. Plastic ingestion is thus an evolutionary trap, a phenomenon that occurs when cues are decoupled from their previously associated high fitness outcomes. Theory predicts that dominance hierarchies could dictate individual responses to evolutionary traps across social environments, but the social dimension of evolutionary trap responses has rarely been investigated. We tested how variation in group size influences the formation of dominance relationships and, in turn, how these dominance relationships drive differences in foraging behavior in Western mosquitofish (*Gambusia affinis*). This included foraging for a variety of familiar and novel food‐like items, including microplastics. Overall, dominant individuals were often the first to sample food and had higher bite rates than subordinates, including when foraging for microplastics. Importantly, how dominance affected foraging behavior depended on group size and on whether groups were presented with familiar or novel foods. Furthermore, individuals were consistent in their foraging behavior across trials with different group sizes, indicating the formation of stable social roles. These results suggest that predicting the ecological and evolutionary consequences of evolutionary traps will require an understanding of how social structures influence trap susceptibility.

## INTRODUCTION

Over 1500 different species, aquatic and terrestrial, have been documented consuming plastic (Santos et al., 2021), a costly behavior with documented impacts on individual health and population dynamics (Galafassi et al., 2021; Lyu et al., 2021; Markic et al., 2020; Tang et al., 2018). As the extent of our planet's plastic pollution problem is uncovered, understanding what causes this behavior is critical for our attempts to mitigate it. Plastic consumption can be understood as an evolutionary trap (Santos et al., 2021), created when cues are decoupled from their previously associated fitness outcome (Pollack, Munson, Savoca, et al., 2022; Robertson et al., 2013; Schlaepfer et al., 2002). Animals can get “trapped” into consuming plastic when nutritive food cues emanate from plastic particles. Evolutionary traps are common and only growing more numerous in the modern world (e.g., billions of insects mistake glass and asphalt for water due to the way these surfaces reflect polarize light; Egri et al., 2017; Robertson & Horváth, 2019; Szaz et al., 2015). While there has been extensive study on interspecific variation in plastic consumption across taxa (Roman et al., 2019; Savoca et al., 2016; Schuyler et al., 2014; Wilcox et al., 2015), research on patterns of intraspecific variation in plastic ingestion is limited (but see Nanninga et al., 2020, 2021; Pollack, Munson, Savoca, et al., 2022; Pollack, Munson, Zepeda, et al., 2022). This leaves open important questions about which individuals within vulnerable populations might be most susceptible to eating plastic and why so that management efforts can most effectively target those individuals and potential long‐term eco‐evolutionary dynamics identified.

Previous studies indicate that both prior experience with plastic (Baird & Hooker, 2000; Coppock et al., 2019) and age (Denuncio et al., 2011; Scherer et al., 2017) drive individual‐level differences in consumption patterns in some species; however, by ignoring the social context in which animals experience traps existing, research might be missing a critical aspect underlying behavioral variation (Pollack, Munson, Zepeda, et al., 2022). In many species, the social environment has major impacts on behavior (Aplin et al., 2015; Cantor et al., 2021; Papageorgiou & Farine, 2020; Strauss & Holekamp, 2019). Dominance hierarchies, in which individuals' position reflects their competitive ability relative to other groups members, are a consistent feature of many animal societies (e.g., Bush et al., 2016; Grosenick et al., 2007; Shizuka & McDonald, 2015; Strauss et al., 2022; Strauss & Holekamp, 2019; Tibbetts & Dale, 2004). Social dominance is often associated with priority access to resources, such as food (Clutton‐Brock et al., 1984; Lee & Cowlishaw, 2017; Robbers et al., 2021) or mates (Chen et al., 2011; Smith, 1993; Wroblewski et al., 2009). Hierarchy position also likely influences foraging behavior because rank is associated with different energetic demands (Castro et al., 2006; Killen et al., 2014). When evolutionary traps involve foraging, as with microplastics, dominance might thus be important in determining who is most susceptible to an evolutionary trap, and why (Sapolsky & Share, 2004).

There is mixed evidence in the literature about whether dominants or subordinates are most at risk. If dominants control preferential access to food sources, then subordinates might be impelled to forage in unfamiliar or riskier contexts and are thus more likely to consume novel foods compared with dominants (Heinrich et al., 1995; Reader & Laland, 2001; Seok An et al., 2011; Stahl et al., 2001). Under this hypothesis, subordinates should be particularly susceptible to an evolutionary trap, since they are more likely to risk consuming nonfood items, especially when foraging around dominant individuals. In contrast, in other scenarios dominant individuals might be more at risk for falling for evolutionary traps (e.g., Sapolsky & Share, 2004). This could be the case if dominants play the social role of initiators within their groups, especially in terms of accessing resources (King et al., 2008; Peterson et al., 2002; Smith et al., 2015). Initiators usually have greater access to encountered food, since arriving first at a food patch allows a greater opportunity to exploit it (Krause et al., 2000). Under this alternative hypothesis, dominants would be expected to consume the greatest quantity of any resource, including microplastics. Similarly, if dominants displace subordinates, even if subordinates get to a food patch first, dominants can still end up consuming more. This could be especially relevant with an evolutionary trap, where a novel item appears like a familiar beneficial resource.

Further complicating these predictions, patterns of dominance are not phylogenetically constrained; groups within the same species can have different dominance network structures (Hobson et al., 2021; Shizuka & McDonald, 2015). Critically, the behavioral and physiological impacts of dominance rank are expected to depend both on the structure of the dominance hierarchy and on the individual's position within that hierarchical network (Varholick et al., 2019; Williamson et al., 2017). For example, who takes on the initiator role might change depending on the context (Brent et al., 2015; Goll et al., 2023; McComb et al., 2011; Nagy et al., 2013). For example, while there is some evidence that individuals maintain consistent leader–follower roles within a group (Nakayama et al., 2012), in some cases, individuals take different social roles depending on the situation (Tuliozi et al., 2021). Even something as simple as changes in overall group size can affect the number of potential dyadic relationships at both the group and individual level, thus altering each individual's social experience.

We conducted a series of controlled experiments with groups of mosquitofish (*Gambusia affinis*) to determine how relative dominance rank modulate social roles and subsequent differences in foraging for both familiar and novel foods, including plastic particles. We hypothesized that dominant individuals would act as initiators in a foraging context, and that this would translate into greater exploitation of food resources compared with subordinates. To test whether the effect of dominance on food access is generalized across variation in dominance network size, we ran identical experiments with different group sizes. To test whether effects of dominance on consumption are consistent across unfamiliar foods that are, and are not, evolutionary traps, we presented fish with a series of potential novel food items that included microplastics.

Specifically, we examined the differences among groupmates across three different group sizes in four behaviors: likelihood to sample first when foraging for either familiar or novel foods and number of bites when foraging for either familiar or novel foods. We quantified (1) individual consistency in these four behaviors and (2) how these behaviors relate to dominance ranks. Lastly, (3) we assessed whether differences between dominance ranks in foraging behavior depended on novel food type.

## METHODS

### Study system

We examined the influence of dominance rank on foraging behaviors in Western mosquitofish (*G. affinis*) in different‐sized social groups. Mosquitofish are one of the most widespread introduced species in the world (Pyke, 2008), being dietary generalists and thriving in human‐dominated landscapes and thus likely encounter many novel items in their environment. Mosquitofish form fission–fusion shoals in the wild of various group sizes and compositions in the wild, including single‐sex groups (Fryxell et al., 2015).

As an evolutionary trap, we presented fish with polyethylene particles since polyethylene is the most common plastic debris (Andrady, 2011). Furthermore, polyethylene adsorbs greater concentrations of toxicants than other common plastics (Rochman et al., 2013). In a prior study, we observed that mosquitofish consume polyethylene beads in the laboratory (Pollack, Munson, Zepeda, et al., 2022) and confirmed that particles were ingested with postmortem dissections (Pollack, unpublished observation). Polyethylene particles were distilled from facewash (XtraCare Oil‐Free Foaming Acne Wash Facial Scrub) and either dried (i.e., virgin plastic) or kept in unfiltered water from Putah Creek (Yolo County, CA) for 1 month to accumulate natural biofilm growth (i.e., biofouled plastics). Misleading cues from the biofouling process can cause plastics to smell like natural food sources (Savoca et al., 2016, 2017), making it a potentially more misleading trap than virgin plastic alone.

### Group formation

Observations of fish groups were performed between August 2019 and April 2020 at the Center for Aquatic Biology and Aquaculture (CABA) facilities at the University of California, Davis. Adult female fish were donated from the Sacramento‐Yolo Mosquito and Vector Control District. Females were individually tagged with Visible Implant Elastomer (VIE) tags (Northwest Marine Technologies) and then randomly sorted into groups of 10 that were housed in 37.8‐L tanks (14:10 light: dark photoperiod, 22°C) for at least 4 months prior to experiments to acclimate to lab conditions. During this period, fish were fed ad libitum with a mixture of fish flakes (Tetramin) and floating pellets (New Life Spectrum).

For the experiment, 156 fish (mean standard length = 28.5 ± 3.2 mm) were randomly assigned to groups of two, three, or four individuals, with 16 replicates of two, 20 replicates of three, and 16 replicates of four. To control for the influence of familiarity on behavior, all fish assigned to a social group were from different housing tanks. Fish were given a 24‐h acclimation period in the observation tank post group formation before observations began. Observation tanks consisted of a 37.8‐L tank with a 15‐cm‐long polyvinyl chloride (PVC) pipe (3.81 cm diameter) refuge and airline tubing through which food items could be introduced to the tank out of the fish's view. Observations were conducted once a day between 0900 and 1800 for 10 successive days to evaluate dominance relationships and quantify foraging behavior.

### Determination of dominance ranks

Observations were recorded after a 5‐min adjustment to the presence of the observer (as in Liss et al., 2020; Lopez et al., 2018). Animals could not be observed blind, since group size treatments were apparent to the observer; however, by using a rotating schedule, observers were blind to previous days' observations. Prior to data collection, observers were trained extensively to ensure consistency between observers. Immediately following the adjustment period, the observer recorded all chasing events, the identity of both the chaser and the chased fish for 5 min. It is standard practice to consider aggression received, in this case chases, as an indicator of subordinate position of social fish in general (Fitzpatrick et al., 2008; Matthews & Wong, 2015).

Daily ranking was determined from average daily Elo scores (EloRating; Neumann et al., 2011). Starting all individuals within a group at the same initial score, the Elo rating method updates scores based on a series of wins and losses in dyadic interactions, with winners increasing their numerical score, while losers decrease in score after each interaction (Albers & de Vries, 2001). Changes in numerical score depend on the probability that a higher scored individual wins, with unexpected outcomes leading to a larger change in score than expected outcomes (Elo, 1978). To account for the dynamic nature of dominance hierarchies, each fish's dominance rank was reevaluated and reassigned each day depending on their updated Elo score, informed by data from all previous days. If ties in score occurred, both individuals were assigned the lower rank (e.g., two individuals tied within a group of 2 were both given a rank of 2, two individuals tied within a group of 3 were both given a rank of 3 if the third individual had a higher score, or both given a rank of 2 if the third individual had a lower score). This allowed for higher ranks to emerge as interactions progressed over the course of the 10‐day observation period. Elo scores can be used to calculate hierarchy stability over a given time period using the ratio of rank changes per individual, providing an index score between 0 (totally unstable) and 1 (no changes in rank) (Neumann et al., 2011). The average stability index across all groups was 0.83.

### Social foraging assays

#### Familiar food assay

Directly after the 5‐min observation period of intragroup aggression, groups were observed for the 5 min immediately following the introduction of familiar floating pellets (New Life Spectrum). To standardize the level of competition, food was scaled for group size such that there were two pellets per fish. The observer would then record the feeding order and number of bites taken of pellets by each fish in the group. Since fish would attempt to try to take a bite from an item or take multiple bites from a single food item, we used bite count as our estimate for rate of consumption.

#### Novel food assay

For the last 5 days of observations, a novel food was introduced to the groups immediately following the daily familiar food assay. Groups were observed for the 5 min immediately following the introduction of novel food through airline tubing. Including novel food only after the groups had been together for 5 days allowed time for the dominance hierarchies to form. The novel food was varied for each day to maintain novelty but introduced in the same sequence across days. If there is a sequence effect where the response to a food type depends on previous experience, then this design does not allow us to rigorously compare relative preference for different novel foods. However, exposing all individuals to the same sequence of food types (thus standardizing any potential sequence effect) allows us to more cleanly test our focal hypotheses about how group size and dominance ranks influence foraging on each novel food. The novel foods introduced were brine shrimp (highly palatable, day 6), glass beads (not palatable, day 7), aspen woods chips (not palatable, day 8), virgin microplastics (potential evolutionary trap, day 9), and biofouled microplastics (potential evolutionary trap, day 10). Novel foods (except for glass beads) were scaled for group size—3 mg per fish (i.e., the same mass per fish as the familiar food pellets). Instead of scaling for weight, glass beads were scaled to count (2 beads per fish). Biofouled microplastics were piped into arenas in a standardized volume of biofouling water (i.e., 0.5 mL) for all group sizes to control for the intensity of the olfactory cue of added stream water. Furthermore, it allowed us to maintain the same ecologically relevant freshwater concentration of microplastics within the assay tanks (~0.05 ppm) (Li et al., 2020). See Appendix S1 for details on source and size of novel items.

All foods were introduced through tubing with a flush of 30 mL of water to limit associations between human handling of food and the introduction of food at the surface of the water. At the conclusion of the last observation day (trial day 10), individuals were weighed and measured for standard length. These procedures were approved by the University California Davis Institutional Animal Care and Use Committee (protocol #19357).

### Statistical analysis

We used Bayesian generalized linear multilevel models, fitted with the R package brms (Bürkner, 2018), to analyze all behavioral data. Out of the 520 trials in this study, 18 were not included in the final data set due to a scheduling error.

In order to evaluate the influence of dominance rank on an individual's likelihood to sample familiar and novel foods first out of the entire group (i.e., initiate foraging for the group), we used a Bernoulli structure. To assess how individual dominance rank affects the count of bites for familiar food, we used a zero‐inflated negative binomial structure, while we used a zero‐inflated Poisson structure for the count of bites for novel food. The zero‐inflated structure allows us to account for the fact that multiple processes could be causing fish to take zero bites during a trial (e.g., fish that were never going to take a bite given infinite time vs. those were prevented from taking a bite by other fish). For all models, daily dominance rank, group size, and trial number were included as predictors. To account for the potential that larger individuals are simply the strongest exploitative competitors (i.e., bigger, faster swimmers) and will thus typically be first and eat more, we included body length as a predictor in all our models. All models included varying intercepts for fish ID nested within group ID, to account for consistent differences among individuals within different groups. For the familiar food models, trial was treated as an integer; however, for the novel food models, trial was treated as a categorical variable to account for differences in novel items. For beta coefficients of fixed effects, we used weakly informative, regularizing priors centered on 0, meaning the models were skeptical of high beta values.

In order to quantify repeatability of behaviors, the intra‐class correlation coefficient (ICC; Nakagawa et al., 2017) was calculated for individual fish ID separately for all the models described above (with unnested varying intercept for fish ID) using the rptR package in R (Stoffel et al., 2017), with 1000 bootstrapping in order to determine 95% CI. For this analysis, a Poisson structure was used to assess the repeatability for the number of both familiar and novel bites (instead of zero‐inflated). To assess whether the same individual initiated foraging in both familiar and novel food contexts, an additional model was run with an individual's likelihood of eating novel food first as the outcome (Bernoulli structure) and whether they ate first during the familiar food trial, group size, and trial as predictors. The model also included varying intercepts for fish ID nested within group ID.

To assess whether the amount of familiar food consumed before the novel trial influenced the number of novel bites taken (i.e., the impact of nutritional state), we included number of familiar food bites as an additional predictor in the model of novel food bites. For groups of two, the model with known food bites fit better using leave‐one‐out cross‐validation (LOO) as a criterion, but not for groups of three or four. To keep model structure identical across groups to allow for comparison, we elected to leave it out for all models.

To derive inference about differences in behaviors between ranks, we used posterior odds ratios to interpret these preplanned contrasts. Posterior odds ratios were derived directly from posterior parameter estimates of the multilevel models described above. For each draw from the posterior, the difference in estimated mean response is calculated for each pairwise combination of ranks, resulting in one value per pair per draw. This allows us to present the mean and credible intervals for each contrast.

## RESULTS

Across all trials, 80% of individuals took at least one bite of familiar food and 79% of individuals took at least one bite of novel food.

### The consistency of individual foraging behaviors

ICC values are reported in Table 1. For familiar food, the repeatabilities of foraging behaviors across all group sizes ranged from 0.20 to 0.38, which is within the range commonly observed in studies of a broad range of behaviors in a broad range of taxa (Bell et al., 2009). In contrast, the number of bites of novel food was not as repeatable, perhaps because novel foods were different between trials.

**TABLE 1:** Intra‐class correlation coefficients (ICC) across several trials assessing how likely mosquitofish individuals are to be the first in their group to sample a food item and the number of bites of that food within a trial.

Behavior	Food type	All groups		Groups of 2		Groups of 3		Groups of 4	
		ICC	95% CI	ICC	95% CI	ICC	95% CI	ICC	95% CI
First to eat	Familiar food	0.26	[0.17–0.33]	0.38	[0.20–0.48]	0.28	[0.16–0.41]	0.20	[0.08–0.32]
	Novel food	0.16	[0.05–0.21]	0.31	[0.05–0.48]	0.14	[0.01–0.23]	0.06	[0.00–0.13]
No. bites	Familiar food	0.33	[0.23–0.38]	0.24	[0.09–0.38]	0.37	[0.24–0.47]	0.31	[0.20–0.41]
	Novel food	0.10	[0.00–0.18]	0.11	[0.00–0.26]	0.08	[0.00–0.18]	0.10	[0.00–0.22]

*Note*: CI is the confidence interval obtained by parametric bootstrapping.

In addition to being consistent across trials, the initiator role was also consistent across assay types within the same day. That is, an individual was more likely to sample first in a novel food trial if they ate first in the familiar food trial that day (estimate = 0.81, 95% CI = 0.39–1.22). See Appendix S2 for all posterior parameter estimates for this analysis.

### Differences in likelihood to sample food first

**FIGURE 1:**
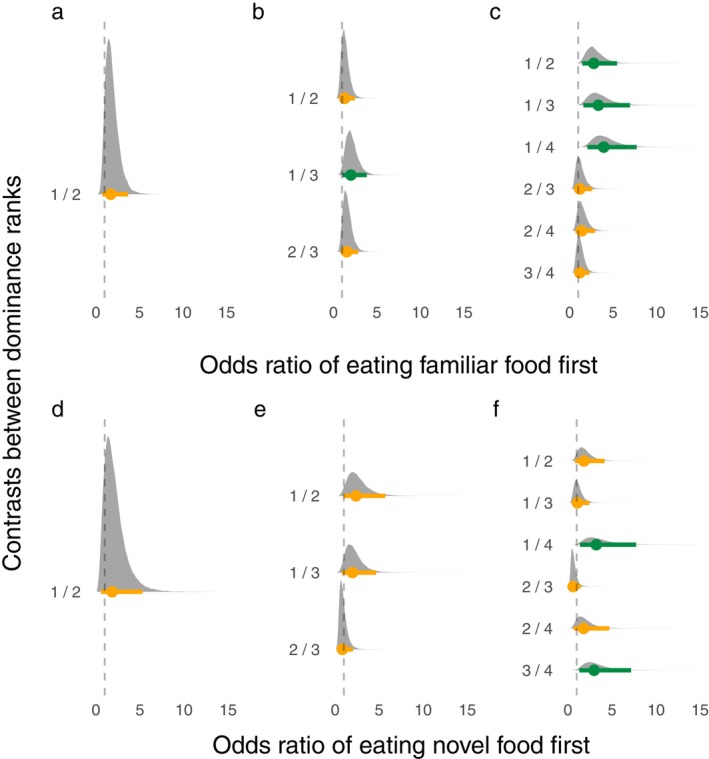
Odds ratio distributions of eating familiar first for (a) groups of two, (b) groups of three, and (c) groups of four based on differences in ranks. Odds ratio distributions of eating novel food first for (d) groups of two, (e) groups of three, and (f) groups of four based on differences in ranks. Odds ratio distributions are derived from the posterior parameter estimates of multilevel models and are estimates of the differences in dominance ranks for their mean likelihood to take a bite first. Dots represent the estimated mean, while lines represent the 95% credible intervals. Orange denotes that the credible intervals overlap with 1, while green denotes that the credible intervals do not overlap 1. Dashed lines indicate when the odds ratio is equal to 1.

Model structure and all posterior parameter estimates, including odds ratios, are reported in Appendix S3. Overall, we found that rank affected an individual's likelihood to sample familiar food first, but only for the highest ranked individuals in the two larger group sizes (Figure 1a–c). That is, for groups of two, differences were not observed between individuals of different rank. However, for groups of three, top ranked individuals were more likely to sample first compared with the lowest ranked individual. Differences were not observed between the second and third ranked individuals. Similarly, for groups of four, the top ranked individual was more likely to sample first compared with all lower ranked individuals. However, differences were not observed between individuals in the lower dominance ranks.

Rank differences did not affect likelihood to sample novel food first, except for the lowest ranked individuals in the largest group sizes (Figure 1d–f). For groups of two and three, rank did not appear to influence feeding order for novel food. For groups of four, there was no observed differences between most ranks. The exception to this pattern was that lowest rank fish were less likely to sample novel food first compared with the second lowest ranked and the highest ranked fish. Length did not affect the likelihood to sample familiar or novel food first.

### Differences in number of bites

**FIGURE 2:**
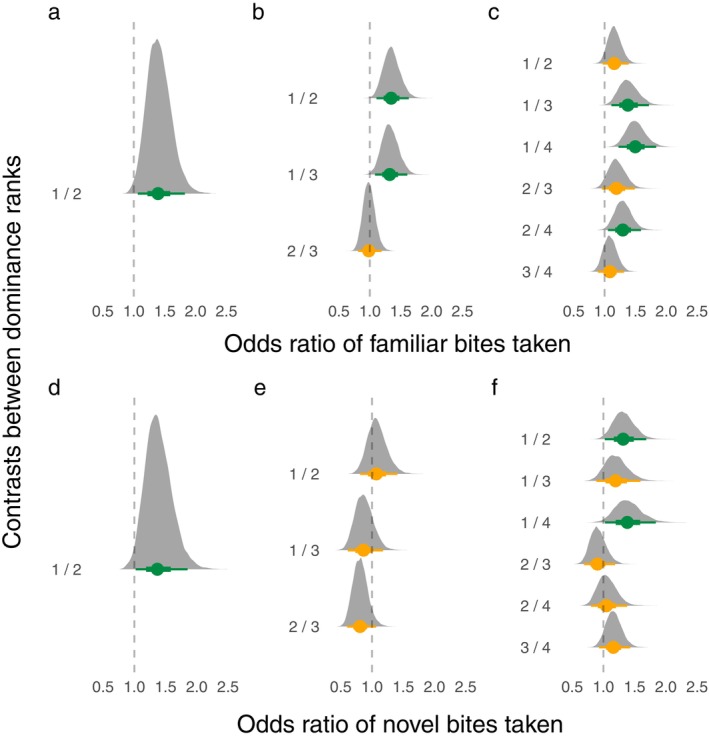
Odds ratio distributions of bites of familiar food taken for (a) groups of two, (b) groups of three, and (c) groups of four. Odds ratio distributions of bites of novel food taken for (d) groups of two, (e) groups of three, and (f) groups of four. Odds ratio distributions are derived from the posterior parameter estimates of the multilevel models and are estimates of the differences in dominance ranks for their mean bite rate. Dots represent the estimated mean, while lines represent the 95% credible intervals. Orange denotes that the credible intervals overlap with 1, while green denotes that the credible intervals do not overlap 1. Dashed lines indicate when the odds ratio is equal to 1.

Model structure and all posterior parameter estimates, including odds ratios, are reported in Appendix S4. Overall, rank affected the number of bites taken of familiar food within the 5‐min trial, although the exact pattern depended on group size (Figure 2a–c). For groups of two, the higher ranked individual took the most bites. For groups of three, the highest ranked individuals took more bites than both lower ranked individuals. However, differences were not observed between the lower ranked individuals. For groups of four, if ranks differed by two or more, the higher ranked individual took more bites, but if the fish had adjacent ranks, rank did not clearly explain difference in number of bites. That is, we are less confident in the difference between ranks 1 and 2, ranks 2 and 3, and ranks 3 and 4 (since the 95% CIs barely overlap zero).

When foraging for novel food, differences between ranks again depended on group size but differed from the patterns observed in the familiar food trials (Figure 2d–f). For groups of two, the highest ranked individuals took the most bites. For groups of three, there were no differences in novel bites between ranks. For groups of four, the highest ranked individuals took more bites than the second highest ranked and lowest ranked individuals. However, differences were not observed between other ranks. Length did not affect the number of bites taken of familiar or novel food.

### Differences in foraging across novel food types

Model structure and posterior parameter estimates are all reported in Appendix S5. We did not observe differences between ranks in likelihood to sample first for any of the novel food types. Differences in number of bites for items based on rank were mostly supported in the largest group size, where either the highest ranked individual took more bites or the lowest ranked individual took fewer bites compared with other ranks in the group (Table 2). This effect was most striking when fish were offered microplastics. When groups of four were presented with microplastics on trial day 9 (virgin) and day10 (biofouled), top ranked individuals took more bites of both plastic types than most other ranks in the group, regardless of whether the plastic was treated or not (i.e., had an additional attractive olfactory cue). When groups of four were presented with brine shrimp on day 6 (i.e., a palatable and nutritious novel food), rank 4 individuals took fewer bites than rank 1 and 3 individuals. When presented with glass beads on day 7 (i.e., a nonpalatable novel item), we observed no differences in bites between ranks, and when offered wood chips, in both groups of three and four, the highest ranked individual took more bites than either the second or third ranked individual.

**TABLE 2:** Odds ratio of novel bites taken between different ranks.

Contrast between ranks		Brine shrimp			Glass beads			Wood chips			Virgin microplastics			Biofouled microplastics		
		Estimate	2.5% CI	97.5% CI	Estimate	2.5% CI	97.5% CI	Estimate	2.5% CI	97.5% CI	Estimate	2.5% CI	97.5% CI	Estimate	2.5% CI	97.5% CI
Group of 2	1 vs. 2	0.95	0.61	1.58	3.5	0.82	21.4	1.29	0.68	2.48	0.55	0.23	1.3	1.36	0.41	4.95
Group of 3	1 vs. 2	1.24	0.86	1.82	1.2	0.65	2.32	**1.84**	**1.02**	**3.28**	1.3	0.7	2.33	1.58	0.9	2.86
	1 vs.3	1.18	0.82	1.73	1.37	0.8	2.34	1.54	0.88	2.74	1.45	0.76	2.72	1.19	0.71	2.01
	2 vs.3	0.95	0.65	1.4	1.14	0.57	2.18	0.84	0.46	1.57	1.12	0.59	2.13	0.75	0.41	1.35
Group of 4	1 vs.2	1.25	0.79	2.03	0.81	0.42	1.54	1.26	0.67	2.47	**2.69**	**1.29**	**5.79**	1.52	0.95	2.49
	1 vs.3	1.01	0.67	1.56	0.6	0.3	1.16	**1.83**	**1.02**	**3.32**	**2.34**	**1.02**	**5.47**	**1.66**	**1.02**	**2.74**
	1 vs.4	**1.69**	**1.09**	**2.7**	1	0.52	1.98	1.79	0.98	3.41	**2.18**	**1**	**4.71**	**2.64**	**1.53**	**4.68**
	2 vs.3	0.81	0.48	1.37	0.75	0.37	1.41	1.45	0.74	2.77	0.87	0.35	2.16	1.09	0.64	1.53
	2 vs.4	1.35	0.78	2.36	1.25	0.65	2.38	1.42	0.73	2.77	0.81	0.34	1.92	1.73	0.97	3.13
	3 vs.4	**1.68**	**1.07**	**2.67**	1.67	0.86	3.45	0.99	0.54	1.84	0.93	0.36	2.38	1.6	0.88	2.89

*Note*: Odds ratio distributions are derived from the posterior parameter estimates of the multilevel models and are estimates of the differences in the median number of bites between individuals. CI is the credible interval from posterior parameter estimates. Values are in bold if the 95% CI did not overlap 1.

## DISCUSSION

In most contexts (5/6 experiments), dominant individuals (i.e., those that had the highest Elo score within their group based on agonistic dyadic interactions) consumed more familiar and novel food items than less dominant groupmates. Thus, our findings suggest that while dominant individuals may benefit from greater access to high quality resources, they may also pay higher costs when exposed to noxious novel foods. In particular, we found that in groups of four fish, dominants took more bites of two different types of microplastics, a costly evolutionary trap, than their subordinate groupmates. These findings suggest that the benefits and costs of social dominance may depend on the novel risks associated with a monopolizable resource, especially if novel items emit similar cues as safe or beneficial familiar objects. Moreover, it suggests that both group size and social structure should be considered when assessing the individuals, populations, and species at greatest risk of an evolutionary trap.

Our work adds to the present understanding of how variation in behavior might drive variation in plastic ingestion. Research on lab‐reared fish suggests that activity level and boldness behavior are positively related to plastic ingestion (Nanninga et al., 2020, 2021). While a previous study of mosquitofish indicated that differences between social groups might also drive differences in foraging for plastics (Pollack, Munson, Zepeda, et al., 2022), this work is the first to demonstrate the potential for differences *within* social groups driven by social relationships. That is, differences in trap susceptibility depend both on the group's traits and on the individual's place within that group.

The effect of dominance rank on foraging behavior depended on group size, indicating that it is crucial to consider both the hierarchy structure and individual position within that structure when formulating hypotheses about the relationship between dominance rank and social role (Amici et al., 2020; Varholick et al., 2019). In the larger groups, we observed a despotic structure for access to familiar resources, where dominants were the first to feed and took more bites than subordinates. In contrast, there were no differences in likelihood to initiate foraging for either familiar or novel foods between dominance ranks within groups of two fish. However, the more dominant individuals still took more bites of familiar and novel foods than their subordinate group mates. In essence, dominant individuals were still outcompeting subordinates for bites, even if they were not the first to begin foraging. One potential explanation is that initiator roles are independent of social dominance, as has been observed in other systems (Bousquet & Manser, 2011; Nagy et al., 2013). Post hoc analysis indicates that across all group sizes, fish that were the first to eat tended to take more bites of both familiar and novel foods (Appendix S6) such that it would be beneficial to be the first to eat in a group of two. Yet, dominance was not correlated with the initiator role in this group size.

Surprisingly, we did not find that subordinates predominantly took the initiator role when novel food was presented. Other studies have found that subordinates show lower neophobia when foraging than dominants, likely because they are forced to accept greater risks due to increased hunger (Heinrich et al., 1995; Reader & Laland, 2001; Stahl et al., 2001). In our study, novel foods were presented immediately after familiar food, which could have exacerbated this differential in response to novel foods if dominants were better fed going into the novel food assay. Contrary to this potential bias, subordinates did not approach novel food first, despite possibly being hungrier. Similarly, studies of other systems have failed to observe differences in approaching novel objects between dominant and subordinates (Amici et al., 2020; Greggor et al., 2016). In fact, in some systems, dominant individuals might even take on more risks or costs to the benefit of subordinates (Chiarati et al., 2012). Critically, rapid environmental change might increase the risks of trying novel items and even obfuscate the risks, benefiting none of the group. This is especially concerning with evolutionary traps like consumption of microplastics, where fitness costs (i.e., the bioaccumulation of toxicants) are decoupled from the immediate behavior (Robertson & Blumstein, 2019; Santos et al., 2021).

Body length did not predict most foraging behaviors, indicating that rank more than size affected exploitation of resource. However, size differences were purposefully kept to a minimum when forming groups in this experiment, and larger differences between group members might reveal stronger size‐based differences (Matthews & Wong, 2015). We did find that size was a strong predictor of number of novel food bites taken. While potentially driven by the greater motivation of larger individuals to consume more food, it was curious that we only observed this in the novel and not known food contexts. This might indicate that in novel situations, traits other than previously established dominance ranks might play an important role in competition. Indeed, neophobia levels may easily shift in many systems, like with seasonal changes in metabolic demand, resource availability, or predation pressure (Brown et al., 2013; Greggor et al., 2016).

In addition to dominance, other factors might drive differences in foraging behaviors between group mates. For example, in a study comparing dominance styles across different species of macaques, Amici et al. (2020) found that centrally located individuals within the group's social network were more likely to approach novel items, but only in less despotic groups. While rank did not influence differences in neophobia, network structure was important for both neophobic behavior and food sharing within these groups of macaques. Furthermore, innate differences in individual behavioral traits may drive variation in social role taking and voraciousness (Nagy et al., 2013; Nakayama et al., 2012). For example, more aggressive individuals often have higher food intake rates (Biro & Stamps, 2008). Similarly, variation in nutritional state could drive differences in willingness to explore novel food options; hungry animals with lower energy reserves might be more motivated to find and consume foods in novel environments (Moran et al., 2021). That is, in some circumstances, intrinsic individual differences might be better predictors of within‐group variation than emergent hierarchical positions. Ultimately, dominance structure and its impact on variation in response to evolutionary traps might be species specific, or even population specific. Nonetheless, to properly assess variation in risk, these features of animal sociality ought to be considered.

Our experimental design brings up some caveats to our interpretation of the results. First, both familiar and novel foods were introduced through the same feeding tube such that fish could have become conditioned to the feeding tube by the time the novel foods were introduced on day 6. However, since the effect of dominance rank on behavior changed between familiar and novel foods, we believe that fish responded to novel foods differently. Furthermore, since novel foods were introduced in the same order for all groups to account for order effects, this could influence how fish responded to subsequent novel foods if prior novel foods were in the recent past highly palatable (i.e., be more accepting of novel foods after receiving the palatable brine shrimp first;Pollack, Munson, Zepeda, et al., 2022). However, even though we observed that dominant fish took more bites of the palatable brine shrimp within groups of four fish, dominants behaved differently toward the highly unpalatable glass beads they received the next day. That is, they appear to have adjusted their behavior accordingly. Thus, by the last 2 days of the experiment, when microplastics were received, fish had experienced both palatable and unpalatable novel foods in their recent past.

Nutritional state could also affect interest in a novel food like plastic particles (Santos et al., 2021). That is, by the time fish received glass beads or microplastics, they were well fed from a series of daily feedings and therefore potentially less interested overall in interacting with novel foods. However, our measure of familiar food ingested (i.e., familiar food bites) did not explain foraging behavior for novel foods in groups of three or four. While it did seem to matter for groups of 2, it was in the opposite direction than expected if nutritional state drove this behavior (Appendix S7). Individuals in groups of two took more bites of novel food if they took more bites of familiar food in the 5 minutes prior. Moreover, familiar food pellets were kept to a minimum such that all pellets were finished by the end of the 5‐min feeding assay. That is, we do not believe any of the fish had hit their satiation limit within the experimental trials, even the most dominant fish.

### Implications for management

Understanding factors and dynamics that explain variation in susceptibility to falling into evolutionary traps can improve management by developing stronger predictions and targeted interventions at the individual level. First, social dynamics can help identify situations where focal organisms are more likely to fall for a trap (i.e., anticipatory management; Mouquet et al., 2015). In species that are known to have strong leader–follower dynamics or common social learning strategies (e.g., many fish, birds, and mammals), it could be particularly useful to identify “leader,” “dominant,” or “keystone” individuals (Modlmeier et al., 2014) for targeted interventions. While other studies have found that individuals can lead group members into evolutionary traps (i.e., Donaldson et al., 2012; Sigaud et al., 2017), this work highlights the interaction between individual and group‐level traits. That is, if group‐level traits (e.g., group size or sex ratio) affect the types of individuals (e.g., dominance rank or personality type) that tend to drive group decision‐making, then these mediating factors should also be identified. Once these individuals within a social system are identified, training “leaders” to avoid falling into traps could prevent entire groups from becoming trapped. While actively teaching enough individuals to achieve this outcome might not be feasible for fission–fusion societies like mosquitofish, it could be manageable for other species with clear leaders in relatively stable social groups.

What about the consumption of plastics more specifically? A previous framework highlighted that in order to identify species or populations most at risk for plastic consumption, researchers should consider the level of physical and chemical resemblance between prey and plastic particles, the selectivity of the species (i.e., are they generalist vs. specialists), the nutritional state of the population (i.e., are they starving), and the relative availability of plastic nearby (Santos et al., 2021). Our results suggest that in our system, and possibly in many social systems, variation in group size and dominance should also be considered when identifying individuals most at risk within a population. Our results also suggest that since individuals in larger groups might be the most susceptible to plastic ingestion, larger groups within a population should be targeted for mitigation efforts before smaller ones. However, it should be noted that since larger groups might be better able to buffer better against the loss of group members, this might counteract issues of high skew in plastic ingestion (Maldonado‐Chaparro & Chaverri, 2021).

Although considering social dynamics might be useful for anticipatory management of plastic consumption, it might not be suitable for learning interventions specifically. That is, the time lag between consumption of plastic and the emergence of fitness costs could make learning to avoid plastics under natural conditions unlikely (Greggor et al., 2019; Santos et al., 2021). Thus, human intervention to decrease exposure to the potential trap has been suggested as the primary method of active mitigation (Santos et al., 2021).

Finally, behaviors can be used as biomonitors for contamination, especially in freshwater contexts (e.g., Bownik & Wlodkowic, 2021; Gerhardt, 2007). Dominant individuals would be a logical starting place when monitoring individuals for pollution levels, since they might bioaccumulate toxicants at a higher rate than subordinates. This would be especially relevant for common, abundant invasive species like mosquitofish that could serve as biomonitors of freshwater plastic levels within local food webs.

### Future directions

Finally, while this experiment explores how variation in the social environment influences responses to environmental change (i.e., novel foods), environmental change also affects social environments in and of itself. For instance, warming temperatures have been linked to changes in aggression patterns and pollution can hinder social communication, likely leading to less stable hierarchies in the short term (Fisher et al., 2021). Increasingly variable environmental conditions might also disrupt dominance hierarchies. For example, groups of three‐spined stickleback had decreased hierarchy stability when exposed to simulated turbulence and drought in laboratory experiments (Sneddon et al., 2006). Furthermore, various environmental changes are often simultaneous, and multiple stressors could have an antagonistic or synergistic effect on aggression patterns and subsequent hierarchy formation (Lopez et al., 2023; Orr et al., 2020). Thus, groups likely encounter novel items and experience multiple stressors simultaneously in a changing world. If, as suggested, these abiotic stressors destabilize dominance hierarchies, then dominants may not necessarily consistently outcompete subordinates for foraging. This might lead to a greater shared cost of consuming an evolutionary trap across all group members, instead of concentrating costs at the top of the hierarchy. Future research on feedbacks between the effects of various stressors on social environments, and the subsequent effects of these changes on responses to other aspects of environmental change is therefore needed.

## AUTHOR CONTRIBUTIONS

Lea Pollack and Andrew Sih designed the research. Lea Pollack performed the experiment. Lea Pollack and Michael Culshaw‐Maurer analyzed and visualized the data. Lea Pollack wrote the manuscript with assistance from all coauthors.

## CONFLICT OF INTEREST STATEMENT

The authors declare no conflicts of interest.

## Supporting information


Appendix S1:

Appendix S2:

Appendix S3:

Appendix S4:

Appendix S5:

Appendix S6:

Appendix S7:

## Data Availability

Data and code (Pollack et al., 2024) are available in Zenodo at https://doi.org/10.5281/zenodo.14291382.
