# Supplementary material for: Social dominance influences individual susceptibility to an evolutionary trap in mosquitofish
Source: Ecol Appl. 2025 Jan 20;35(1):e3081. doi: 10.1002/eap.3081 (PMC11744343; doi:10.1002/eap.3081)
Supplement: Supplementary file 1 — Appendix S1: [file EAP-35-e3081-s007.pdf]

Appendix S1. Novel Foods

Title: Social dominance influences individual susceptibility to an evolutionary trap in mosquitofish
Authors: Lea Pollack, Michael Culshaw-Maurer, and Andrew Sih
Journal: Ecological Applications

Appendix S1: Table S1. Items for novel food assay.

Novel Item	Trial	Source	Approximate Size	Image
Brine Shrimp	6	Omega One Freeze Dried Brine Shrimp	0.1 – 3 mm	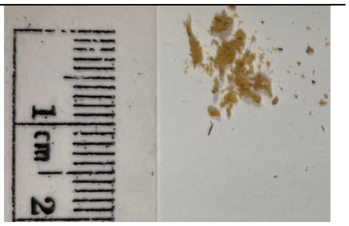
Glass Beads	7	“Seed Beads”, manufacturer unknown	1 – 4 mm	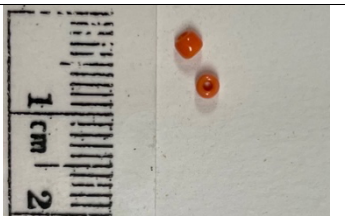
Wood Chips	8	Zoo Med Aspen Snake Bedding	0.5 – 4 mm	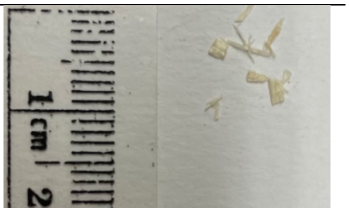
Microplastics (Polyethylene)	9 & 10	XtraCare Oil-Free Foaming Acne Wash Facial Scrub	0.1 – 1 mm	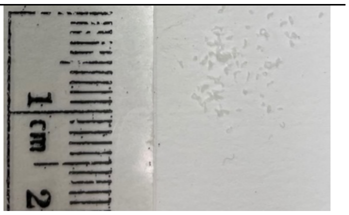

Photography credit for all images: L. Pollack.
