# Supplementary material for: Social dominance influences individual susceptibility to an evolutionary trap in mosquitofish
Source: Ecol Appl. 2025 Jan 20;35(1):e3081. doi: 10.1002/eap.3081 (PMC11744343; doi:10.1002/eap.3081)
Supplement: Supplementary file 2 — Appendix S2: [file EAP-35-e3081-s001.pdf]

**Appendix S2. Likelihood to sample novel food first across all group sizes**

**Title:** Social dominance influences individual susceptibility to an evolutionary trap in mosquitofish
**Authors:** Lea Pollack, Michael Culshaw-Maurer, and Andrew Sih
**Journal:** Ecological Applications

Appendix S2**:** Table S1. Posterior parameter estimates for model of likelihood to be first to sample novel food across all group sizes as predicted by whether an individual ate familiar food first during that day's trial.

| parameter | estimate | 2.5% | 97.5% |
|---|---|---|---|
| intercept | 0.37 | -0.79 | 1.51 |
| first to sample familiar food | 0.81 | 0.39 | 1.22 |
| group size | -0.48 | -0.82 | -0.15 |
| trial 6 vs.7 | -0.40 | -0.92 | 0.12 |
| trial 6 vs.8 | -0.01 | -0.52 | 0.50 |
| trial 6 vs.9 | -0.06 | -0.58 | 0.46 |
| trial 6 vs.10 | -0.03 | -0.55 | 0.50 |

Appendix S2**:** Table S2. Median odds ratios for the model of likelihood to be first to sample novel food across all group sizes as predicted by whether an individual ate familiar food first during that day's trial.

| Contrast between trials | estimate | 2.5% | 97.5% |
|---|---|---|---|
| brine vs.biofouled | 1.03 | 0.609 | 1.73 |
| brine vs.bead | 1.49 | 0.883 | 2.52 |
| brine vs.pine | 1.01 | 0.608 | 1.69 |
| brine vs.virgin | 1.06 | 0.633 | 1.79 |
| bead vs.biofouled | 0.692 | 0.395 | 1.22 |
| bead vs.pine | 0.679 | 0.398 | 1.16 |
| bead vs.virgin | 0.716 | 0.404 | 1.26 |
| pine vs.biofouled | 1.02 | 0.588 | 1.77 |
| pine vs.virgin | 1.05 | 0.612 | 1.83 |
| virgin vs.biofouled | 0.967 | 0.551 | 1.68 |

*Contrasts are calculated from posterior parameter estimate quantile intervals for each trial.*

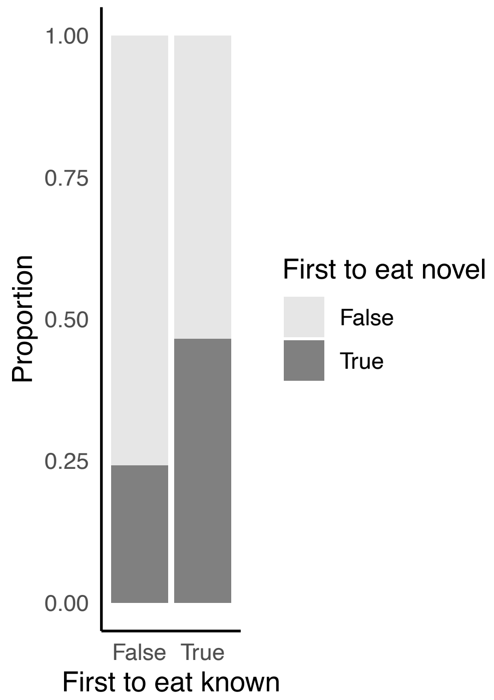


Appendix S2**:** Figure S1. Proportion of individuals who ate first for both familiar and novel food during the same day's trials.
