# Supplementary material for: Social dominance influences individual susceptibility to an evolutionary trap in mosquitofish
Source: Ecol Appl. 2025 Jan 20;35(1):e3081. doi: 10.1002/eap.3081 (PMC11744343; doi:10.1002/eap.3081)
Supplement: Supplementary file 3 — Appendix S3: [file EAP-35-e3081-s006.pdf]

Appendix S3. Likelihood to sample familiar and novel foods for all ranks

Title: Social dominance influences individual susceptibility to an evolutionary trap in mosquitofish
Authors: Lea Pollack, Michael Culshaw-Maurer, and Andrew Sih
Journal: Ecological Applications

Appendix S3**:** Table S1. Model structure and posterior parameter estimates for all models of familiar bite order.

Model Structure	Posterior parameter estimates for fixed effects			
	parameter	estimate	2.5% CI	97.5% CI
**Likelihood to sample familiar food first for group of 2** ~ 1 + daily rank + length + trial + (1| group / fish ID)	intercept	-0.14	-1.23	0.90
	daily rank 1 vs. 2	-0.54	-1.32	0.25
	trial	0.05	-0.05	0.15
	length	-0.04	-0.76	0.67
**Likelihood to sample familiar food first for group of 3** ~ 1 + daily rank + length + trial + (1| group / fish ID)	intercept	-0.53	-1.32	0.23
	daily rank 1 vs. 2	-0.27	-0.91	0.37
	daily rank 1 vs. 3	-0.70	-1.35	-0.06
	trial	-0.01	-0.08	0.07
	length	0.08	-0.33	0.49
**Likelihood to sample familiar food first for group of 4** ~ 1 + daily rank + length + trial + (1| group / fish ID)	intercept	-0.36	-1.15	0.41
	daily rank 1 vs. 2	-1.03	-0.69	-0.38
	daily rank 1 vs. 3	-1.20	-1.93	-0.46
	daily rank 1 vs. 4	-1.37	-2.04	-0.72
	trial	-0.02	-0.11	0.06
	length	0.17	-0.30	0.65

Appendix S3**:** Table S2. Median odds ratios of contrasts between ranks for each model of likelihood to be first to sample familiar food.

**contrast between ranks**		**estimate**	**2.5%CI**	**97.5% CI**
Group of 2	1 vs.2	1.71	0.77	3.69
Group of 3	1 vs.2	1.31	0.69	2.47
	1 vs.3	2.02	1.06	3.84
	2 vs.3	1.54	0.84	2.83
Group of 4	1 vs.2	2.79	1.65	4.83
	1 vs.3	3.31	1.82	6.07
	1 vs.4	3.93	2.32	6.79
	2 vs.3	1.19	0.61	2.27
	2 vs.4	1.40	0.77	2.56
	3 vs.4	1.19	0.69	2.03

Contrasts are calculated from posterior parameter estimate quantile intervals for each rank.

Appendix S3**:** Table S3. Model structure and posterior parameter estimates for all models of novel bite order.

Model Structure	Posterior parameter estimates for fixed effects			
	parameter	estimate	2.5% CI	97.5% CI
**Likelihood to sample novel food first for group of 2** ~ 1 + daily rank + length + trial + (1| group / fish ID)	intercept	0.32	-0.89	1.47
	daily rank 1 vs. 2	-0.59	-1.64	0.53
	trial 6 vs. 7	-0.75	-1.76	0.24
	trial 6 vs. 8	-0.06	-1.03	0.91
	trial 6 vs. 9	-0.06	-1.05	0.92
	trial 6 vs. 10	0.09	-0.90	1.09
	length	-0.37	-1.22	0.44
**Likelihood to sample novel food first for group of 3** ~ 1 + daily rank + length + trial + (1| group / fish ID)	intercept	-0.34	-1.19	0.45
	daily rank 1 vs. 2	-0.85	-1.72	0.05
	daily rank 1 vs. 3	-0.67	-1.54	0.25
	trial 6 vs. 7	-0.34	-1.12	0.44
	trial 6 vs. 8	-0.06	-0.86	0.73
	trial 6 vs. 9	-0.14	-0.92	0.64
	trial 6 vs. 10	0.04	-0.72	0.82
	length	0.05	-0.35	0.44
**Likelihood to sample novel food first for group of 4** ~ 1 + daily rank + length + trial + (1| group / fish ID)	intercept	-0.75	-1.54	0.00
	daily rank 1 vs. 2	-0.60	-1.43	0.22
	daily rank 1 vs. 3	-0.08	-0.89	0.75
	daily rank 1 vs. 4	-1.17	-2.06	-0.29
	trial 6 vs. 7	-0.20	-0.99	0.56
	trial 6 vs. 8	-0.01	-0.77	0.73
	trial 6 vs. 9	-0.03	-0.86	0.79
	trial 6 vs. 10	-0.14	-0.99	0.68
	length	-0.18	-0.62	0.25

Appendix S3**:** Table S4. Median odds ratios of contrasts between ranks for each model of likelihood to be first to sample novel food

**contrast between ranks**		**estimate**	**2.5%CI**	**97.5% CI**
Group of 2	1 vs.2	1.85	0.58	5.30
Group of 3	1 vs.2	2.38	1.13	4.81
	1 vs.3	1.98	0.94	3.99
	2 vs.3	0.83	0.41	1.72
Group of 4	1 vs.2	1.82	0.94	3.56
	1 vs.3	1.08	0.55	2.08
	1 vs.4	3.22	1.60	6.57
	2 vs.3	0.60	0.28	1.23
	2 vs.4	1.78	0.81	3.93
	3 vs.4	2.97	1.51	6.03

Contrasts are calculated from posterior parameter estimate quantile intervals for each rank.
