# Supplementary material for: Social dominance influences individual susceptibility to an evolutionary trap in mosquitofish
Source: Ecol Appl. 2025 Jan 20;35(1):e3081. doi: 10.1002/eap.3081 (PMC11744343; doi:10.1002/eap.3081)
Supplement: Supplementary file 4 — Appendix S4: [file EAP-35-e3081-s003.pdf]

Appendix S4. Number of familiar and novel bites for all ranks

Title: Social dominance influences individual susceptibility to an evolutionary trap in mosquitofish
Authors: Lea Pollack, Michael Culshaw-Maurer, and Andrew Sih
Journal: Ecological Applications

Appendix S4**:** Table S1. Model structure and posterior parameter estimates for all models of familiar food bites.

Model Structure	Posterior parameter estimates for fixed effects			
	parameter	estimate	2.5% CI	97.5% CI
**Familiar food bites for group of 2** ~ 1 + daily rank + length + trial + (1| group / fish ID) **zero inflated** ~ 1 + daily rank + length + trial + (1| group / fish ID)	zero inflated intercept	-8.40	-13.95	-4.29
	zero inflated daily rank 1 vs. 2	3.20	0.22	7.17
	zero inflated length	-0.87	-3.35	1.19
	zero inflated trial	0.25	-0.11	0.66
	intercept	1.26	0.81	1.69
	daily rank 1 vs. 2	-0.33	-0.61	-0.06
	trial	-0.02	-0.05	0.02
	length	-0.17	-0.44	0.07
	shape	4.74	3.05	7.27
**Familiar food bites for group of 3** ~ 1 + daily rank + length + trial + (1| group / fish ID) **zero inflated** ~ 1 + daily rank + length + trial + (1| group / fish ID)	zero inflated intercept	-13.34	-23.98	-7.44
	zero inflated daily rank 1 vs. 2	7.57	2.39	17.68
	zero inflated daily rank 1 vs. 3	7.37	2.12	17.54
	zero inflated length	-0.10	-1.15	0.80
	zero inflated trial	0.37	0.13	0.69
	intercept	1.20	0.97	1.43
	daily rank 1 vs. 2	-0.30	-0.49	-0.11
	daily rank 1 vs. 3	-0.28	-0.47	-0.08
	trial	-0.03	-0.05	-0.01
	length	-0.01	-0.13	0.11
	shape	38.43	10.64	139.71
**Familiar food bites for group of 4** ~ 1 + daily rank + length + trial + (1| group / fish ID) **zero inflated** ~ 1 + daily rank + length + trial + (1| group / fish ID)	zero inflated intercept	-10.22	-25.55	1.06
	zero inflated daily rank 1 vs. 2	-0.19	-20.07	17.77
	zero inflated daily rank 1 vs. 3	10.36	1.48	32.22
	zero inflated daily rank 1 vs. 4	8.97	0.51	28.61
	zero inflated length	0.96	-1.18	3.98
	zero inflated trial	-0.78	-1.68	-0.30
	intercept	1.41	1.16	1.66
	daily rank 1 vs. 2	-0.15	-0.33	0.04
	daily rank 1 vs. 3	-0.32	-0.54	-0.10
	daily rank 1 vs. 4	-0.40	-0.61	-0.20
	trial	-0.05	-0.07	-0.03
	length	0.13	-0.02	0.28
	shape	15.60	7.43	36.50

Appendix S4**:** Table S2. Median odds ratios of contrasts between ranks for each model of familiar food bites.

contrast between ranks		estimate	2.5%CI	97.5% CI
Group of 2	1 vs.2	1.39	1.06	1.83
Group of 3	1 vs.2	1.35	1.11	1.63
	1 vs.3	1.32	1.08	1.61
	2 vs.3	0.98	0.81	1.19
Group of 4	1 vs.2	1.16	0.95	1.38
	1 vs.3	1.38	1.10	1.71
	1 vs.4	1.50	1.20	1.80
	2 vs.3	1.19	0.95	1.47
	2 vs.4	1.29	1.05	1.59
	3 vs.4	1.08	0.89	1.31

Contrasts are calculated from posterior parameter estimate quantile intervals for each rank.

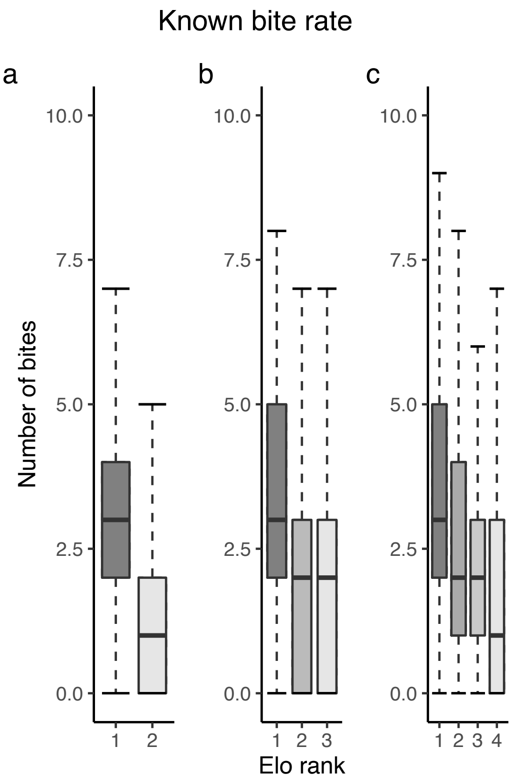

Appendix S4**:** Figure S1. Raw count data for bites of familiar food for (a) groups of 2, (b) groups of 3, and (c) groups of 4. Box plots include the mean latency and interquartile range (IQR) with whiskers extenting to +/1 1.5 IQR. Outliers have been removed from these plots to improve visualization.

Appendix S4**:** Table S3. Model structure and posterior parameter estimates for models of novel food bites.

Model Structure	Posterior parameter estimates for fixed effects			
	parameter	estimate	2.5% CI	97.5% CI
**Novel food bites for group of 2** ~ 1 + daily rank + length + trial + (1| group / fish ID)<br><br>**zero inflated** ~ 1 + daily rank + length + trial + (1| group / fish ID)	zero inflated intercept	-8.45	-21.85	-3.39
	zero inflated daily rank 1 vs. 2	0.99	-1.23	2.90
	zero inflated length	-0.49	-2.04	0.57
	zero inflated trial 6 vs. 7	8.48	3.23	22.10
	zero inflated trial 6 vs. 8	4.08	-1.20	17.22
	zero inflated trial 6 vs. 9	6.05	1.22	19.41
	zero inflated trial 6 vs. 10	5.86	1.02	19.07
	intercept	1.85	1.52	2.19
	daily rank 1 vs. 2	-0.31	-0.61	-0.02
	trial 6 vs. 7	0.94	0.73	1.15
	trial 6 vs. 8	-0.10	-0.32	0.11
	trial 6 vs. 9	-0.22	-0.45	0.00
	trial 6 vs. 10	-0.11	-0.33	0.12
	length	0.02	-0.23	0.26
**Novel food bites for group of 3** ~ 1 + daily rank + length + trial + (1| group / fish ID)<br><br>**zero inflated** ~ 1 + daily rank + length + trial + (1| group / fish ID)	zero inflated intercept	-12.09	-26.65	-5.43
	zero inflated daily rank 1 vs. 2	3.12	0.67	8.68
	zero inflated daily rank 1 vs. 3	2.88	0.47	8.52
	zero inflated length	-0.09	-0.84	0.57
	zero inflated trial 6 vs. 7	8.80	3.00	22.59
	zero inflated trial 6 vs. 8	6.66	0.96	20.20
	zero inflated trial 6 vs. 9	6.58	0.52	20.11
	zero inflated trial 6 vs. 10	5.50	-0.54	19.06
	intercept	1.36	1.08	1.62
	daily rank 1 vs. 2	-0.07	0.14	-0.34
	daily rank 1 vs. 3	0.15	-0.17	0.49
	trial 6 vs. 7	0.01	-0.20	0.20
	trial 6 vs. 8	0.85	0.69	1.00
	trial 6 vs. 9	-0.50	-0.72	-0.28
	trial 6 vs. 10	-0.11	-0.30	0.07
	**length**	**0.17**	**0.03**	**0.31**
**Novel food bites for group of 4** ~ 1 + daily rank + length + trial + (1| group / fish ID)<br><br>**zero inflated** ~ 1 + daily rank + length + trial + (1| group / fish ID)	zero inflated intercept	-10.02	-22.92	-3.83
	zero inflated daily rank 1 vs. 2	0.31	-1.62	2.09
	zero inflated daily rank 1 vs. 3	1.86	0.06	3.95
	zero inflated daily rank 1 vs. 4	1.55	-0.24	3.58
	zero inflated length	0.86	-0.05	2.00
	zero inflated trial 6 vs. 7	7.98	2.22	20.96
	zero inflated trial 6 vs. 8	5.74	0.12	18.55
	zero inflated trial 6 vs. 9	5.63	-0.22	18.61
	zero inflated trial 6 vs. 10	5.48	-0.42	18.49
	intercept	1.36	1.07	1.64
	daily rank 1 vs. 2	-0.28	-0.52	-0.03
	daily rank 1 vs. 3	-0.18	-0.48	0.12
	daily rank 1 vs. 4	-0.33	-0.62	-0.03
	trial 6 vs. 7	0.33	0.14	0.52
	trial 6 vs. 8	0.93	0.77	1.09
	trial 6 vs. 9	-0.14	-0.35	0.08
	trial 6 vs. 10	-0.15	-0.37	0.07
	length	0.16	-0.02	0.34

Appendix S4**:** Table S4. Median odds ratios of contrasts between ranks for each model of novel food bites.

**contrast between ranks**		**estimate**	**2.5%CI**	**97.5% CI**
Group of 2	1 vs.2	1.37	1.02	1.85
Group of 3	1 vs.2	1.07	0.81	1.41
	1 vs.3	0.86	0.62	1.18
	2 vs.3	0.81	0.60	1.07
Group of 4	1 vs.2	1.32	1.07	1.61
	1 vs.3	1.19	0.94	1.51
	1 vs.4	1.38	1.09	1.75
	2 vs.3	0.91	0.73	1.13
	2 vs.4	1.05	0.84	1.31
	3 vs.4	1.16	0.97	1.37

Contrasts are calculated from posterior parameter estimate quantile intervals for each rank.

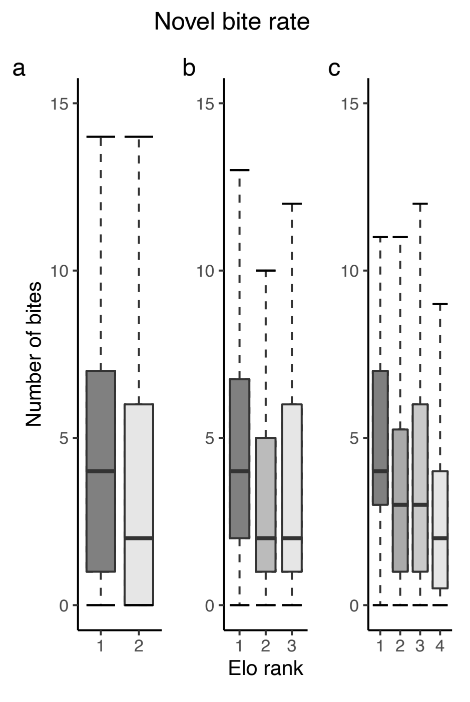

Appendix S4**:** Figure S2. Raw count data for bites of novel food for (a) groups of 2, (b) groups of 3, and (c) groups of 4. Box plots include the mean latency and interquartile range (IQR) with whiskers extenting to +/1 1.5 IQR. Outliers have been removed from these plots to improve visualization.
