# Supplementary material for: Social dominance influences individual susceptibility to an evolutionary trap in mosquitofish
Source: Ecol Appl. 2025 Jan 20;35(1):e3081. doi: 10.1002/eap.3081 (PMC11744343; doi:10.1002/eap.3081)
Supplement: Supplementary file 5 — Appendix S5: [file EAP-35-e3081-s004.pdf]

Appendix S5. Differences between ranks in foraging for specific novel foods

Title: Social dominance influences individual susceptibility to an evolutionary trap in mosquitofish
Authors: Lea Pollack, Michael Culshaw-Maurer, and Andrew Sih
Journal: Ecological Applications

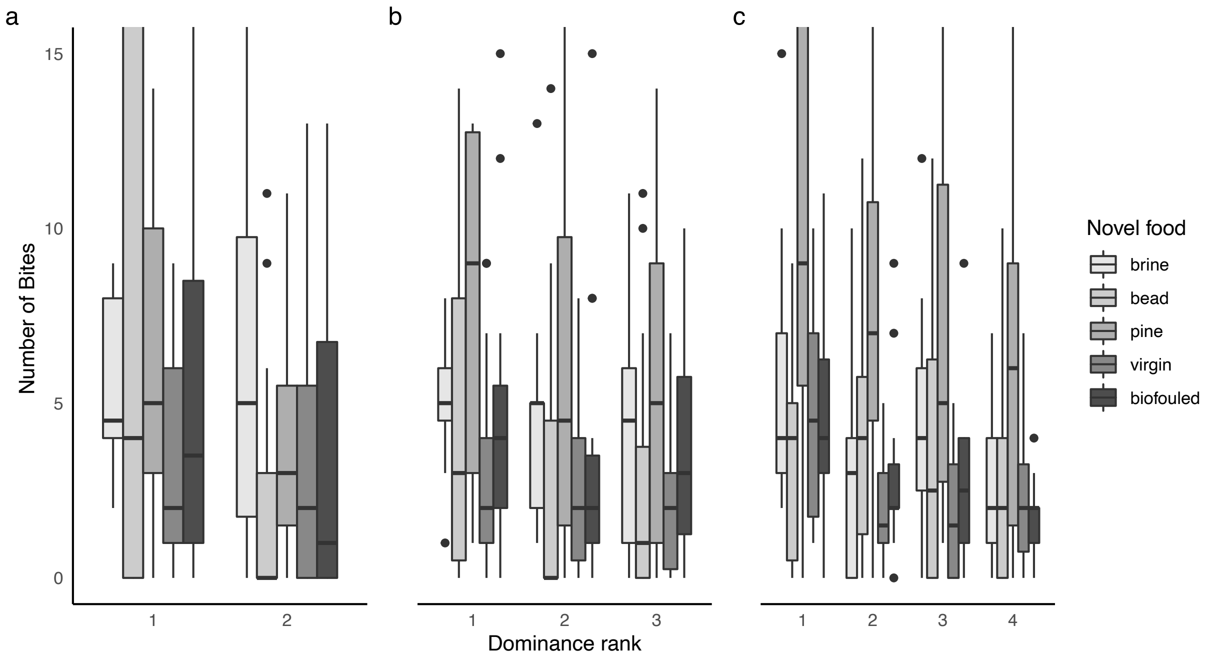

Appendix S5**:** Figure S1. Raw count data for bites of novel food for (a) groups of 2, (b) groups of 3, and (c) groups of 4. Box plots include the mean latency and interquartile range (IQR) with whiskers extenting to +/1 1.5 IQR. Plots have been zoomed in on the y-axis to improve visualization of differences, not all outliers are shown.

Appendix S5**:** Table S1.Posterior parameter estimates for all models of number of bites for each novel food type organized by group size.

		**Number of novel food bites**								
		brine shrimp			glass bead			pine chips		
	parameter	estimate	2.5%	97.5%	estimate	2.5%	97.5%	estimate	2.5%	97.5%
**Group of 2**	intercept	1.7	1.2	2.1	2.1	0.3	3.3	1.7	1.2	2.2
	zero inflated intercept	-6.1	-13.7	-1.8	-3	-12.6	1.9	-5	-12.1	-1.2
	daily rank 1 vs. 2	0	-0.5	0.5	-1.3	-3.1	0.2	-0.3	-0.9	0.4
	zero inflated rank 1 vs. 2	3	-2.1	10.9	4.1	-4.3	19.8	2.5	-2.4	9.9
**Group of 3**	intercept	1.6	1.3	1.8	1.3	0.5	1.9	2	1.4	2.6
	zero inflated intercept	-7.2	-16.3	-2.7	-6.8	-24.9	-0.4	-7.5	-16.4	-2.7
	daily rank 1 vs. 2	-0.2	-0.6	0.2	-0.2	-0.8	0.4	-0.6	-1.2	0
	daily rank 1 vs. 3	-0.2	-0.5	0.2	-0.3	-0.8	0.2	-0.4	-1	0.1
	zero inflated rank 1 vs. 2	0.1	-9.9	10.3	11	0.5	51.5	2.6	-7	12.3
	zero inflated rank 1 vs. 3	3.1	-4.5	12.7	4	-4.7	22	3.5	-4.7	13.1
**Group of 4**	intercept	1.6	1.3	1.9	1.3	0.8	1.8	2.3	1.8	2.8
	zero inflated intercept	-9.6	-22.6	-2.8	-9.7	-33.4	0.8	-5.9	-16.5	-1.5
	daily rank 1 vs. 2	-0.2	-0.7	0.2	0.2	-0.4	0.9	-0.2	-0.9	0.4
	daily rank 1 vs. 3	0	-0.4	0.4	0.5	-0.2	1.2	-0.6	-1.2	0
	daily rank 1 vs. 4	-0.5	-1	-0.1	0	-0.7	0.7	-0.6	-1.2	0
	zero inflated rank 1 vs. 2	8.4	1.1	21.7	0.6	-12.2	25.3	2.6	-4.4	13.7
	zero inflated rank 1 vs. 3	5.9	-2.4	19.2	20.1	-0.3	67.7	-2.6	-15.1	9.6
	zero inflated rank 1 vs. 4	6.1	-1.9	19.5	9.4	-3.9	40.5	2.7	-4.1	13.8

		Number of novel food bites					
		virgin microplastic			biofouled microplastic		
	parameter	estimate	2.5%	97.5%	estimate	2.5%	97.5%
**Group of 2**	intercept	1.1	0.3	1.7	1.4	0.4	2.2
	zero inflated intercept	-5.7	-13.7	-1.2	-5.3	-13.1	-0.9
	daily rank 1 vs. 2	0.6	-0.3	1.5	-0.3	-1.6	0.9
	zero inflated rank 1 vs. 2	5.5	0.1	15.2	3.8	-2.5	13
**Group of 3**	intercept	1	0.5	1.5	1.3	0.8	1.7
	zero inflated intercept	-4	-11.6	-0.8	-6.7	-15.3	-2.3
	daily rank 1 vs. 2	-0.3	-0.8	0.4	-0.5	-1	0.1
	daily rank 1 vs. 3	-0.4	-1	0.3	-0.2	-0.7	0.3
	zero inflated rank 1 vs. 2	-0.7	-9.9	7.8	1.9	-8.1	11.5
	zero inflated rank 1 vs. 3	0.1	-7.8	8.6	1.4	-7.4	10.6
**Group of 4**	intercept	1.5	0.9	2	1.4	0.8	1.9
	zero inflated intercept	-8.2	-20.6	-2.3	-0.4	-0.9	0
	daily rank 1 vs. 2	-1	-1.8	-0.2	-0.5	-1	0
	daily rank 1 vs. 3	-0.8	-1.7	0	-1	-1.5	-0.4
	daily rank 1 vs. 4	-0.8	-1.5	0	0.6	0.3	1.3
	zero inflated rank 1 vs. 2	1.8	-10.8	16.1	0.2	0	0.6
	zero inflated rank 1 vs. 3	4.7	-7.1	19	0.1	-0.6	0.8
	zero inflated rank 1 vs. 4	3.5	-9.4	17.9	-0.1	-0.7	0.6

Appendix S5**:** Table S2. Posterior parameter estimates for all models of likelihood to sample first for each novel food type organized by group size.

		**Likelihood to sample novel food first**								
		brine shrimp			glass bead			wood chips		
	parameter	estimate	2.5%	97.5%	estimate	2.5%	97.5%	estimate	2.5%	97.5%
**Group of 2**	intercept	0.2	-1.7	2	-0.3	-1.9	1.2	0.4	-1.5	2.0
	daily rank 1 vs. 2	-0.3	-2.0	1.5	-0.9	-2.4	0.8	-0.9	-2.5	1.0
**Group of 3**	intercept	-0.7	-2.2	0.5	-1.3	-2.8	-0.1	0.1	-1.1	1.1
	daily rank 1 vs. 2	-0.3	-1.8	1.4	-0.3	-1.8	1.2	-1.4	-2.6	0.0
	daily rank 1 vs. 3	-0.7	-2.2	0.9	0.0	-1.5	1.5	-1.5	-2.8	-0.2
**Group of 4**	intercept	-1.7	-3	-0.6	-1.8	-3.1	-0.8	-1.1	-2.6	0.1
	daily rank 1 vs. 2	0.0	-1.5	1.4	0.5	-1.0	1.9	-0.4	-1.8	1.2
	daily rank 1 vs. 3	1.1	-0.3	2.5	1.1	-0.2	2.5	-0.5	-1.9	1.0
	daily rank 1 vs. 4	0.1	-1.3	1.5	-0.4	-1.9	1.0	-0.7	-2.1	0.8

		**Likelihood to sample novel food first**					
		virgin microplastic			biofouled microplastic		
	parameter	estimate	2.5%	97.5%	mean	2.5%	97.5%
**Group of 2**	intercept	0.2	-1.6	1.9	0.3	-1.6	2.1
	daily rank 1 vs. 2	-0.5	-2.3	1.3	-0.6	-2.3	1.3
**Group of 3**	intercept	-1	-2.6	0.3	-0.5	-2	0.6
	daily rank 1 vs. 2	-0.1	-1.6	1.5	-1.3	-2.7	0.2
	daily rank 1 vs. 3	-0.3	-1.8	1.3	-0.2	-1.5	1.2
**Group of 4**	intercept	-0.6	-1.9	0.5	-1	-2.5	0.2
	daily rank 1 vs. 2	-1.1	-2.5	0.4	-0.5	-2	1.1
	daily rank 1 vs. 3	-0.4	-1.8	1	-0.5	-1.9	1.1
	daily rank 1 vs. 4	-1.4	-2.9	0.1	-0.8	-2.4	0.8

Appendix S5**:** Table S3. Median odds ratios of contrasts between ranks for each model of likelihood to sample novel food first, broken down by novel food type.

		Brine shrimp			Glass beads			Wood chips		
contrast between ranks		estimate	2.5%	97.5%	estimate	2.5%	97.5%	estimate	2.5%	97.5%
Group of 2	1 vs.2	1.33	0.22	7.3	2.4	0.44	11.3	2.44	0.38	12.2
Group of 3	1 vs.2	1.3	0.26	5.98	1.39	0.3	6.01	3.93	0.99	13.8
	1 vs.3	1.99	0.39	8.67	1.04	0.23	4.37	4.71	1.25	16.6
	2 vs.3	1.5	0.21	10.9	0.74	0.12	4.57	1.2	0.26	5.86
Group of 4	1 vs.2	0.97	0.24	4.46	0.61	0.15	2.61	1.45	0.31	6.19
	1 vs.3	0.31	0.08	1.37	0.32	0.09	1.24	1.66	0.35	6.9
	1 vs.4	0.89	0.23	3.57	1.47	0.36	6.4	1.99	0.44	8.17
	2 vs.3	0.33	0.05	2.04	0.52	0.09	2.73	1.14	0.17	7.72
	2 vs.4	0.92	0.15	5.15	2.4	0.39	14	1.38	0.2	9.23
	3 vs.4	2.81	0.5	15.7	4.61	0.88	25.5	1.2	0.19	7.47

		Virgin microplastics			Biofouled microplastics		
contrast between ranks		estimate	2.5%	97.5%	estimate	2.5%	97.5%
Group of 2	1 vs.2	1.76	0.28	9.65	1.82	0.27	9.72
Group of 3	1 vs.2	1.07	0.22	5.04	3.74	0.8	15.4
	1 vs.3	1.31	0.28	5.84	1.17	0.29	4.44
	2 vs.3	1.22	0.16	9.09	0.32	0.05	1.85
Group of 4	1 vs.2	2.85	0.65	12.7	1.61	0.34	7.37
	1 vs.3	1.5	0.35	5.87	1.6	0.35	6.96
	1 vs.4	4.18	0.92	18.6	2.29	0.47	10.5
	2 vs.3	0.52	0.08	3.2	0.99	0.14	6.68
	2 vs.4	1.45	0.22	9.56	1.43	0.2	10.3
	3 vs.4	2.81	0.43	18.3	1.44	0.2	9.93

Contrasts are calculated from posterior parameter estimate quantile intervals for each rank.
