# Supplementary material for: Social dominance influences individual susceptibility to an evolutionary trap in mosquitofish
Source: Ecol Appl. 2025 Jan 20;35(1):e3081. doi: 10.1002/eap.3081 (PMC11744343; doi:10.1002/eap.3081)
Supplement: Supplementary file 6 — Appendix S6: [file EAP-35-e3081-s005.pdf]

Appendix S6. The influence of feeding order on number of bites

Title: Social dominance influences individual susceptibility to an evolutionary trap in mosquitofish
Authors: Lea Pollack, Michael Culshaw-Maurer, and Andrew Sih
Journal: Ecological Applications

Appendix S6**:** Table S1. Model structure and posterior parameter estimates for all models of familiar food bites.

**Model Structure**	**Posterior parameter estimates for fixed effects**			
	parameter	estimate	2.5% CI	97.5% CI
**Familiar food bites for group of 2** ~ 1 + first to eat + length + trial + (1| group / fish ID)<br><br>**zero inflated** ~ 1 + first to eat + length + trial + (1| group / fish ID)	zero inflated intercept	-1.44	-7.08	2.46
	zero inflated first to eat	-9.80	-18.86	-4.93
	zero inflated trial	0.09	-0.37	0.88
	zero inflated length	-0.34	-2.57	1.34
	intercept	0.96	0.54	1.37
	**first to eat**	**0.40**	**0.12**	**0.16**
	trial	-0.03	-0.07	0.01
	length	-0.17	-0.43	0.09
	shape	6.07	3.71	9.80
**Familiar food bites for group of 3** ~ 1 + first to eat + length + trial + (1| group / fish ID)<br><br>**zero inflated** ~ 1 + first to eat + length + trial + (1| group / fish ID)	zero inflated intercept	-5.08	-8.14	-2.74
	zero inflated first to eat	-7.34	-15.49	-3.29
	zero inflated trial	0.33	0.11	0.60
	zero inflated length	-0.45	-1.30	0.27
	intercept	0.88	0.69	1.07
	first to eat	**0.32**	**0.18**	**0.46**
	trial	-0.02	-0.04	0
	length	0.01	-0.10	0.13
	shape	46.74	12.21	167.05
**Familiar food bites for group of 4** ~ 1 + first to eat + length + trial + (1| group / fish ID)<br><br>**zero inflated** ~ 1 + first to eat + length + trial + (1| group / fish ID)	zero inflated intercept	-2.35	-5.72	1.74
	zero inflated first to eat	-11.31	-23.32	-4.54
	zero inflated trial	-0.60	-1.15	-0.17
	zero inflated length	0.29	-1.84	2.51
	intercept	0.92	0.73	1.11
	**first to eat**	**0.53**	**0.40**	**0.66**
	trial	-0.03	-0.05	-0.01
	length	0.16	0.03	0.3
	shape	56.98	14.57	196.63

Appendix S6**:** Table S2. Model structure and posterior parameter estimates for all models of novel food bites.

Model Structure	Posterior parameter estimates for fixed effects			
	parameter	estimate	2.5% CI	97.5% CI
**Novel food bites for group of 2** ~ 1 + first to eat + length + trial + (1| group / fish ID)<br><br>**zero inflated** ~ 1 + first to eat + length + trial + (1| group / fish ID)	zero inflated intercept	-3.67	-9.32	0.62
	zero inflated first to eat	-7.33	-13.99	-3.59
	zero inflated trial	0.40	-0.13	1.05
	zero inflated length	-0.50	-1.77	0.47
	intercept	2.14	1.19	3.09
	**first to eat**	**0.89**	**0.48**	**1.30**
	trial	-0.11	-0.23	0.01
	length	0.12	-0.09	0.32
	shape	1.65	1.10	2.40
**Novel food bites for group of 3** ~ 1 + first to eat + length + trial + (1| group / fish ID)<br><br>**zero inflated** ~ 1 + first to eat + length + trial + (1| group / fish ID)	zero inflated intercept	-5.03	-12.57	-0.22
	zero inflated first to eat	-6.8	-15.56	-2.3
	zero inflated trial	0.24	-0.32	0.95
	zero inflated length	0.42	-0.78	1.96
	intercept	1.85	1.12	2.57
	**first to eat**	**0.41**	**0.16**	**0.67**
	trial	-0.06	-0.15	0.03
	length	0.19	0.06	0.32
	shape	1.67	1.23	2.23
**Novel food bites for group of 4** ~ 1 + first to eat + length + trial + (1| group / fish ID)<br><br>**zero inflated** ~ 1 + first to eat + length + trial + (1| group / fish ID)	zero inflated intercept	0.10	-5.02	4.65
	zero inflated first to eat	-7.21	-17.9	-2.12
	zero inflated trial	-0.31	-0.98	0.26
	zero inflated length	-0.03	-0.99	0.99
	intercept	1.91	1.13	2.66
	**first to eat**	**0.56**	**0.31**	**0.83**
	trial	-0.07	-0.16	0.03
	length	0.17	0.01	0.33
	shape	2.13	1.49	2.98
