# Supplementary material for: Social dominance influences individual susceptibility to an evolutionary trap in mosquitofish
Source: Ecol Appl. 2025 Jan 20;35(1):e3081. doi: 10.1002/eap.3081 (PMC11744343; doi:10.1002/eap.3081)
Supplement: Supplementary file 7 — Appendix S7: [file EAP-35-e3081-s002.pdf]

Appendix S7. The influence of familiar food bites on novel food bites

Title: Social dominance influences individual susceptibility to an evolutionary trap in mosquitofish
Authors: Lea Pollack, Michael Culshaw-Maurer, and Andrew Sih
Journal: Ecological Applications

Appendix S7**:** Table S1. Model structure and posterior parameter estimates for model of novel food bites with familiar food bites as an additional predictor for groups of 2 fish
.

**Model Structure**	**Posterior parameter estimates for fixed effects**			
	parameter	estimate	2.5% CI	97.5% CI
**Novel food bites for group of 2** ~ 1 + daily rank + familiar bites + length + trial + (1| group / fish ID)<br><br>**zero inflated** ~ 1 + daily rank + familiar bites + length + trial + (1| group / fish ID)	zero inflated intercept	-5.20	-18.98	-0.71
	zero inflated daily rank 1 vs. 2	0.21	-1.43	1.71
	zero inflated familiar bites	-0.67	-1.20	-0.25
	zero inflated length	-0.29	-1.15	0.44
	zero inflated trial 6 vs. 7	6.82	2.49	20.37
	zero inflated trial 6 vs. 8	3.58	-0.89	17.17
	zero inflated trial 6 vs. 9	5.05	0.72	18.67
	zero inflated trial 6 vs. 10	5.01	0.71	18.51
	intercept	1.44	1.11	1.76
	daily rank 1 vs. 2	-0.10	-0.38	0.18
	trial 6 vs. 7	0.48	0.24	0.71
	trial 6 vs. 8	-0.06	-0.27	0.15
	trial 6 vs. 9	-0.26	-0.50	-0.03
	trial 6 vs. 10	-0.10	-0.32	0.14
	**familiar bites**	**0.13**	**0.10**	**0.15**
	length	0.08	-0.14	0.30
